# Interarm Difference in Blood Pressure: Reproducibility and Association with Peripheral Vascular Disease

**DOI:** 10.1155/2014/841542

**Published:** 2014-01-29

**Authors:** Jesper Mehlsen, Niels Wiinberg

**Affiliations:** ^1^Coordinating Research Centre, Frederiksberg Hospital, Nordre Fasanvej 57, 2000 Frederiksberg, Denmark; ^2^Department of Clinical Physiology and Nuclear Medicine, Frederiksberg Hospital, Nordre Fasanvej 57, 2000 Frederiksberg, Denmark

## Abstract

The present study aimed at examining the interarm difference in blood pressure and its use as an indicator of peripheral arterial disease (PAD). Data were included from consecutive patients referred from their general practitioner to our vascular laboratory for possible PAD aged 50 years or older without known cardiac disease, renal disease, or diabetes mellitus. 824 patients (453 women) with mean age of 72 years (range: 50–101) were included. 491 patients had a diagnosis of hypertension and peripheral arterial disease (PAD) was present in 386 patients. Systolic blood pressure was 143 ± 24 mmHg and 142 ± 24 mmHg on the right and left arm, respectively (*P* = 0.015). The interarm difference was greater in patients with hypertension (*P* = 0.002) and PAD (*P* < 0.0005). 443 patients were measured on two separate occasions and the interarm difference for systolic blood pressure was reproducible for differences >20 mmHg. This study confirmed the presence of a systematic but clinically insignificant difference in systolic blood pressure between arms. The interarm difference was larger in hypertension and PAD. Consistent lateralisation is present for differences ≥20 mmHg and an interarm difference >25 mmHg is a reliable indicator of PAD in the legs.

## 1. Introduction

Hypertension is a prevalent condition affecting more than one-third of the adult population in the developed world. Accordingly, measurement of blood pressure in the clinical setting is probably second to none with respect to frequency of recordings and medical consequences resulting from the measurements obtained. A number of concepts regarding technique and cut-off values for the diagnosis of hypertension have evolved, have been tested over more than a century, and have gradually become part of consensus reports and guidelines. Most recommendations on blood pressure measurements and hypertension [1–4] have stated that blood pressure should be measured in both arms and that the arm with the highest value should be used for subsequent measurements. The recent European Guideline on Hypertension [1] gives a more precise description of this by stating that “in the event of a significant (>10 mmHg) and consistent SBP difference between arms…the arm with the higher BP values should be used.” One of the potential problems in these recommendations lies in the reproducibility of standard arm blood pressure readings as pointed out by Stergiou et al. [5] showing that clinical blood pressure measurements had a standard deviation of differences between two sets of measurements of 10.4 mmHg, systolic. Physiological variations and inaccuracies in the technique employed would in itself give rise to a certain random variation of blood pressure readings between the two arms, especially if the measurements are carried out sequentially. Another potential problem with the guideline statement is that according to the recent literature [6] stems from the fact that even though an interarm blood pressure difference above 10 to 15 mmHg is associated with peripheral arterial disease, low sensitivities hamper the use of these cut-off values in screening for cardiovascular disease.

The present study was aimed at a reappraisal of the possible use of an interarm difference in blood pressure as an indicator of peripheral vascular disease. In order to meet this aim, we examined data from our vascular laboratory of blood pressure measured simultaneously on both arms in a large cohort of patients and compared the results to the presence or absence of peripheral arterial disease. We used simultaneous measurements with semiautomatic, oscillometric devices to avoid possible observer bias and we studied the reproducibility of the interarm blood pressure difference in a large subgroup of patients referred for a second set of measurements.

## 2. Methods

### 2.1. Study Population

This was a retrospective observational study using data obtained from a cohort of consecutive patients aged 50 years or older referred from their general practitioner to our vascular laboratory for possible peripheral arterial disease (PAD). None of the patients had a diagnosis of ischaemic heart disease or renal disease (ICD-10 classes I20-25 and N00-19, resp.). None of the patients had been diagnosed with diabetes mellitus (ICD-10 class E10-11) at the time of examination.

### 2.2. Blood Pressure Measurements

Arm blood pressure was measured simultaneously on both arms three times after at least 5 minutes of rest in the supine position using two automated oscillometric devices (Omron 705C, Omron, Japan) and the devices were used at random for the right and left arm. The devices used have passed the validation process defined by the European Society of Hypertension [7]. Ankle blood pressure was measured by mercury-in-silastic strain-gauge plethysmography (DM2000, Medimatic, Denmark) twice with the lower end of the cuff placed about 3 cm above the malleoli and with the cuff wrapped in a cylindrical fashion perpendicularly to the axis of the leg [8, 9]. The strain gauge was placed either on the first toe or on the forefoot depending on the quality of the signal. Ankle brachial index (ABI) was derived by dividing the systolic blood pressure on the ankle by the systolic blood pressure on the upper arm with the highest reading. Definite PAD was regarded to be present if the ABI was less than 0.9 in one leg or both legs. Possible media sclerosis of the arteries at the ankle level was considered at an ABI of 1.3 or higher. A definite normal outcome was considered present when the ABI was equal to or higher than 1.0 and less than 1.3. Patients were classified as having hypertension according to information provided by the general practitioner. The patients were on their usual medication and studies were performed at room temperature between 8 a.m. and 2 p.m. A number of patients were referred twice and had their blood pressure measurements repeated allowing us to examine the reproducibility of the interarm difference in systolic blood pressure. 

### 2.3. Statistical Analysis

Data are given as mean values with standard deviations unless otherwise indicated. Comparisons were made both for the absolute values and for the numerical difference between the two sides. All analyses were carried out using SPSS Statistics 19 (IBM Company, 2010). Comparisons were made with the Student's *t*-test or the chi-squared test when appropriate, using a five per cent two-sided significance level. Predictive values of positive and negative test (i.e., the likelihood of having/not having PAD, resp., at a given interarm difference for systolic blood pressure) using interarm differences in systolic blood pressure as a diagnostic test for PAD were calculated for values of 10, 15, 20, and 25 mmHg, respectively.

## 3. Results 

A total of 824 patients (453 women) with a mean age of 72 years (range: 50–101 years) were included. Systolic blood pressure on arms and ankles is given in Table 1. Systolic blood pressure on the two arms was 143 ± 24 mmHg and 142 ± 24 mmHg on the right and left arm, respectively (*P* = 0.015). Group mean value of systolic blood pressure recorded from the arm with the highest reading was 148 ± 24 mmHg. The differences in systolic blood pressure between the two arms were normally distributed with a mean value of 1.0 ± 11.7 mmHg (*P* = 0.015 for right versus left arm) giving 95% confidence limits of ÷21.9 to +23.9 mmHg. The numerical difference in systolic blood pressure (Table 1) exceeded 10 mmHg in 27.1%, 15 mmHg in 13.2%, 20 mmHg in 6.6%, and 25 mmHg in 3.8% of the total population studied.

Hypertension was present in 491 patients (59.6%) and in these, the numerical blood pressure difference between arms exceeded 10 mmHg in 31.6%, 15 mmHg in 16.5%, 20 mmHg in 7.9%, and 25 mmHg in 5.3%. The numerical differences found in the hypertensive subpopulation were significantly higher than in those from the subgroup without this diagnosis (*P* = 0.002).

The systolic ankle blood pressure varied between 0 and 290 mmHg and the group mean value of systolic blood pressure recorded from the ankles with the lowest readings was 129 ± 43 mmHg. The mean ankle brachial index (ABI) was 0.89 ± 0.29. The prevalence of ABI values in the different classes is given in Table 1. Definite PAD (ABI < 0.9) was present in 396 patients (48.1%), whereas a definite normal result was found in 314 (38.1%), and possible media sclerosis was found in 41 (5.0%) leaving 73 patients (8.8%) who could not be classified with certainty according to the criteria given by current guidelines [9].

The mean numerical interarm difference in systolic blood pressure was higher in patients with definite PAD (9.9 ± 10.5 mmHg) compared to non-PAD patients (6.8 ± 5.6 mmHg, *P* < 0.0005). In patients with definite PAD, the numerical systolic blood pressure difference between arms exceeded 10 mmHg in 32.1%, 15 mmHg in 18.1%, 20 mmHg in 11.1%, and 25 mmHg in 6.7%. These values were significantly higher than in non-PAD patients (*P* < 0.0005). When patients were stratified with respect to the presence of both PAD and hypertension, the distribution of differences in arm blood pressure only differed significantly between PAD and non-PAD patients in the hypertensive group (*P* = 0.013).

We could not demonstrate significant correlations between interarm differences in systolic blood pressure and age neither for the whole group, for those without PAD, nor for those free of both PAD and hypertension.

Using a difference in arm blood pressure as an indicator of PAD resulted in the highest negative predictive value for a difference smaller than 10 mmHg (PV_neg_ = 0.58) and in the highest positive predictive value for a difference greater than 25 mmHg (PV_pos_ = 0.81).

The subgroup, in which arm and ankle pressures were measured on two occasions, included 443 patients with a mean age of 74 ± 9.3 years. The mean time period between measurements was 21.3 ± 17.7 months. Systolic arm blood pressure on the right side was 147 ± 24 mmHg and 146 ± 23 mmHg on the two occasions and 146 ± 24 mmHg and 145 ± 23 mmHg on the left side. The numerical differences between the two sides were 8.4 ± 8.8 mmHg and 8.4 ± 8.6 mmHg, respectively. Patients were allocated into three categories based on interarm difference at the first visit: (1) ≤10 mmHg, (2) >10 mmHg and ≤20 mmHg, and (3) >20 mmHg. A reproducible difference was found in 75.7% of cases at the second visit in category 1, in 27.0% in category 2, and in 41.2% in category 3, respectively. When all patients were included, the lateralisation of the interarm difference was consistent (*P* = 0.004); however, this consistency disappeared for interarm differences of 20 mmHg or less (*P* = 0.052).

## 4. Discussion

This study has shown that systolic blood pressure is slightly higher in the right than in the left arm and that the pressure differs significantly more between the arms in patients with PAD than in those without. It has also shown that this dissimilarity in arm blood pressure only seems to be present in the hypertensive subgroup. In spite of this, the confidence limits of blood pressure differences in normal subjects are of a magnitude that renders this difference imprecise as a diagnostic tool in PAD. 

Five previous studies have analysed possible differences in blood pressure between arms using similar simultaneous measurements as in the present study [10–14], and in a subsequent meta-analysis [15]of the initial four studies, the mean prevalence was 19.6 per cent for differences in systolic arm blood pressure exceeding 10 mmHg (95% CI 18.0–21.3%) and 4.2 per cent for differences exceeding 20 mmHg (95% CI 3.4–5.1%). The fifth study [14] showed that the interarm difference decreased progressively as the number of blood pressure readings increased and only in two out of 145 primarily hypertensive patients did they find a large and consistent interarm difference and both subjects had previously been diagnosed with peripheral arterial disease. Our study is in agreement with two previous studies demonstrating a higher prevalence of interarm differences in hypertensive patients [10] and in patients with known cardiovascular disease [12], whereas the study by Lane et al. [13] did not find any relation between interarm difference and the presence of hypertension, diabetes mellitus, or previous cardiovascular disease. This apparent dissimilarity could possibly be ascribed to the low mean age of participants and the low prevalence of the mentioned conditions in the latter study. The interarm difference was found to be age-dependent by two of the previous studies [12, 13], but not in ours. This dissimilarity could be ascribed to the fact that the previous studies included a larger age range with the youngest being 18 years old.

A recent meta-analysis [6] found an interarm difference of 15 mmHg or more to be associated with peripheral vascular disease at a relative risk ratio of 2.5, but with a mean sensitivity of 15 per cent and a mean specificity of 96 per cent. Assuming a prevalence of peripheral vascular disease of 12 per cent and the specificity and sensitivity reported, an interarm difference of 15 mmHg or more would have a predictive value of a positive test of 34 per cent which would be inadequate for selecting patients for aggressive risk management or medical intervention. It would, however, be useful in selecting patients for further diagnostic procedures such as measurement of carotid intima media thickness or ankle blood pressure in order to establish a more firm ground for intervention. 

We found interarm blood pressure differences to have a low reproducibility with significant lateralization only for differences above 20 mmHg. The poor consistency of differences over time is in line with data reported by Kleefstra et al. [16] in patients with type-2 diabetes.

Differences in blood pressure between arms may have a number of causes such as subclavian artery stenosis, aortic aneurism, aortic coarctation, vasculitis, fibromuscular hyperplasia, connective tissue disorders, and thoracic outlet compression. The overall impression, though, is that the most common diagnostic entity would be subclinical atherosclerosis as suggested by the increased likelihood of finding an interarm difference in hypertension and peripheral arterial disease. This suggestion lends support to the WHO guidelines [2] in which it is recommended to measure the blood pressure in both arms at first visit if there is evidence of PAD.

It has been suggested that the interarm differences could be used for diagnostic purposes in suspected PAD, but based on our findings, this arm difference has to be greater than 20 mmHg in order to be reproducible and greater than 25 mmHg to attain a sufficiently high positive predictive value. According to our calculations, the negative predictive value does not become sufficiently high even at low interarm differences to suggest that the absence of an arm difference could exclude the presence of PAD.

### 4.1. Limitations

The main limitation lies in the fact that the study is of a retrospective character. However, the technique described has been the standard in our laboratory for a number of years and the staff has vast experience in blood pressure measurements and analysis. We are therefore convinced that the results obtained are of a quality that matches those that would be obtained in a prospective study.

The patient group included were relatively old and were referred under the suspicion of PAD. Nevertheless, this group would most likely be the target in screening for PAD in general practice and thus a relevant population for the questions posed.

## 5. Conclusions

Our study has confirmed others in the notion that there is a statistical but no clinical significant difference in blood pressure between arms. Our study has also shown that the interarm difference is greater in hypertensive subjects and in patients with peripheral arterial disease. We have shown that the lateralisation of blood pressure was consistent only for differences of 20 mmHg or more. Finally, we have demonstrated that an interarm difference in blood pressure could be used as an indicator of peripheral arterial disease if the difference is greater than 25 mmHg.

## Figures and Tables

**Table 1:** Systolic blood pressure levels and ankle brachial indices.

Systolic arm blood pressure, right (mmHg)	143 ± 24
Systolic arm blood pressure, left (mmHg)	142 ± 24*
Num. diff. in systolic arm blood pressure (mmHg)	8.3 ± 9.1
Systolic ankle blood pressure, right (mmHg)	139 ± 41
Systolic ankle blood pressure, left (mmHg)	138 ± 41
Ankle brachial index >1.30 (%)	5.0
Ankle brachial index 1.00–1.29 (%)	38.1
Ankle brachial index 0.90–0.99 (%)	8.8
Ankle brachial index 0.40–0.89 (%)	43.7
Ankle brachial index <0.39 (%)	4.4

The table shows systolic blood pressure on both arms and ankles and the numerical difference in systolic blood pressure between the two arms given as mean values ± standard deviations. Percentages of patients were grouped according to their ankle brachial index (ABI). *P = 0.015 for the differences in systolic blood pressure between the two arms.
